# Sevoflurane alleviates hepatic ischaemia/reperfusion injury by up‐regulating miR‐96 and down‐regulating FOXO4

**DOI:** 10.1111/jcmm.16063

**Published:** 2021-06-01

**Authors:** Binghua He, Fan Yang, Yingxia Ning, Yalan Li

**Affiliations:** ^1^ Jinan University Guangzhou China; ^2^ Department of Anesthesiology the Central Hospital of Shaoyang Shaoyang China; ^3^ Department of Gynecology and Obstetrics The First Affiliated Hospital of Guangzhou Medical University Guangzhou China; ^4^ Department of Anesthesiology the First Affiliated Hospital of Jinan University Guangzhou China

**Keywords:** apoptosis, forkhead box protein O4, hepatic ischaemia, microRNA‐96, proliferation, reperfusion injury

## Abstract

Hepatic ischaemia/reperfusion (I/R) injury represents an event characterized by anoxic cell death and an inflammatory response, that can limit the treatment efficacy of liver surgery. Ischaemic preconditioning agents such as sevoflurane (Sevo) have been highlighted to play protective roles in hepatic I/R injury. The current study aimed to investigate the molecular mechanism underlying the effects associated with Sevo in hepatic I/R injury. Initially, mouse hepatic I/R injury models were established *via* occlusion of the hepatic portal vein and subsequent reperfusion. The expression of forkhead box protein O4 (FOXO4) was detected using reverse transcription quantitative polymerase chain reaction and Western blot analysis from clinical liver tissue samples obtained from patients who had previously undergone liver transplantation, mouse I/R models and oxygen‐deprived hepatocytes. The morphology of the liver tissues was analysed using haematoxylin‐eosin (HE) staining, with apoptosis detected *via* TUNEL staining. Immunohistochemistry methods were employed to identify the FOXO4‐positive cells. Mice with knocked out FOXO4 (FOXO4‐KO mice) were subjected to I/R. In this study, we found FOXO4 was highly expressed following hepatic I/R injury. After treatment with Sevo, I/R modelled mice exhibited an alleviated degree of liver injury, fewer apoptotic cells and FOXO4‐positive cells. FOXO4 was a target gene of miR‐96. Knockdown of FOXO4 could alleviate hepatic I/R injury and decrease cell apoptosis. Taken together, the key observations of our study suggest that Sevo alleviates hepatic I/R injury by means of promoting the expression of miR‐96 while inhibiting FOXO4 expression. This study highlights the molecular mechanism mediated by Sevo in hepatic I/R injury.

## 1 INTRODUCTION

Hepatic ischaemia/reperfusion (I/R) injury represents an adverse molecular event often taking place in the context of liver surgery and has the potential to cause liver failure.^1^ Moreover, such a complication during liver surgery has been implicated in elevated mortality associated with dysfunctional liver function.^2^ As hepatic I/R progresses, it causes disordered cellular metabolism and liver inflammation.^3^ A notable inflammatory response and oxidative stress have been well documented in the pathogenesis of hepatic I/R.^4^ Although significant efforts have been invested in the research of hepatic I/R pathogenesis with the view of identifying an effective therapeutic approach for hepatic IR, the molecular mechanisms underlying hepatic I/R remain elusive.^5^ As a widely employed anaesthetic agent, sevoflurane (Sevo) has been reported to provide a protective effect in the setting of I/R organ conditions.^6^ Sevo has been speculated to effectively prevent hepatic I/R injury by promoting antioxidant defence effectors and down‐regulating liver enzymes in connection with microRNAs (miRNAs or miRs).^7^

MicroRNAs represent small non‐coding RNAs that possess regulatory properties in the biological processes such as cellular apoptosis and proliferation.^8^ Accumulating evidence has clarified the protective effect of miRNAs against hepatic I/R injury.^9^, ^10^ Furthermore, as a vertebrate conserved miRNA, miR‐96 has been implicated in a wide array of diseases.^11^ A previous report has suggested that miR‐96 exerts a protective function in neonatal rats against hypoxia/ischaemia‐induced brain injury.^12^ miR‐96 is a liver‐specific miRNA that has been suggested as a potential biomarker of liver injury (mainly through apoptosis, necrosis and necroptosis) or hepatitis with its varied expression highlighted in acute/chronic liver fibrosis and hepatocellular carcinoma.^13^ The up‐regulation of miR‐96‐5p has been reported to ameliorate oxidative stress and hepatic steatosis, thus alleviating non‐alcoholic fatty liver disease.^14^ However, its role in hepatic I/R injury as well as the relationship between Sevo and miR‐96 remains poorly understood. miR‐96 binding to forkhead box O (FOXO) family has been widely investigated, with existing literature suggesting that it influences the progression and development of certain human diseases.^15^, ^16^ The presence of binding sites between miR‐96 and forkhead box protein O4 (FOXO4) was predicted by the ENCORI website (http://starbase.sysu.edu.cn/) in the current study. As a member of the FOXO family, FOXO4 has been reported to influence biological events such as cellular apoptosis, cell cycle and oxidative stress responses through transcriptional activities.^17^ Existing literature has indicated that FOXO4 functions as a promoter in the progression of bile duct ligation‐induced liver injury.^18^ Nonetheless, owing to the scarcity of understanding in regard to the impact of FOXO4 on hepatic I/R injury, the current study aimed to elucidate the molecular mechanism by which Sevo provides protection against hepatic I/R injury by altering the expression of miR‐96 and FOXO4 in a mouse hepatic I/R injury model as well as a hypoxia‐re‐oxygenation (H/R) cell model.

## 2 METHODS AND MATERIALS

### 2.1 Bioinformatics analysis

Data regarding the potential targeting relationship and possible binding sites between miR‐96 and FOXO4 were predicted by the bioinformatics analysis website ENCORI (http://starbase.sysu.edu.cn/).

### 2.2 Sample collection

Human I/R liver tissue samples were obtained from 12 patients who had undergone a hepatectomy procedure due to hepatocellular carcinoma or hepatic cyst at the First Affiliated Hospital of Jinan University between June 2012 and December 2016. Liver biopsies from 12 healthy donors who were unsuitable for liver transplantation due to non‐hepatic reasons served as the control samples.

### 2.3 Animal treatment

Male C57BL/6 mice (n = 190; aged 9 weeks; weighed 16‐24 g) and FOXO4 knockdown mice (as FOXO4‐KO mice; n = 20) (Shanghai Model Organisms Center Inc) were housed under a specific controlled pathogen‐free environment at the First Affiliated Hospital of Jinan University. The mice were subjected to a 12‐h light/dark cycle with controlled temperature (21°C) and humidity (60 ± 5%) and granted free access to food and water. Prior to modelling, the mice were deprived of food but not water.

### 2.4 Establishment of mouse hepatic I/R injury models

A total of 190 C57BL/6 mice were subjected to varying treatments with 12 mice placed in each group. The mice were subjected to I/R and sham operation in line with previously reported methods.^7^, ^19^ The mice were subjected to I/R alone or hepatic portal vein occlusion for 60 minutes with reperfusion for 0, 1, 3, 6, 12 and 24 hours. The sham‐operated mice were treated in accordance with the aforementioned method, with the exception of hepatic portal vein occlusion. The control mice (wild‐type [WT] mice), 24 hours before I/R operation, with 50 mg/kg miR‐96 antagomir administered to the mice via injection, followed by ischaemia for 60 minutes and reperfusion for 24 hours.

The mice subsequently underwent I/R operation (hepatic portal vein occlusion for 60 minutes with reperfusion for 24 hours) and were treated with 2% Sevo (air mixed with Sevo), 50 mg/kg antagomir negative control (NC) and 50 mg/kg miR‐96 antagomir, alone or in combination.

Thirty FOXO4‐KO mice were subjected separately to a sham operation, I/R or I/R together with miR‐96 antagomir (24 hours before I/R operation, 50 mg/kg miR‐96 antagomir was injected to the mice, followed by ischaemia for 60 minutes and reperfusion for 24 hours) with 10 mice in each treatment group, after the anaesthesia by the intramuscular injection of 80 mg/kg ketamine hydrochloride (Ketalar Parke‐Davis; Eczacibasi).^7^ Ten per cent povidone iodine solution (Betadine) was applied to the shaven skin for cleansing purposes. The portal vein, hepatic artery and bile duct were subsequently occluded using a microvascular clip. The clamp was removed after partial hepatic ischaemia for a specified amount of time prior to the start of varying reperfusion time‐points. At the end of this period, the mice were euthanized via exsanguination. The blood samples were collected by cardiac puncture and centrifuged at 3000 *g* for 10 minutes for serum extraction purposes and subsequently stored at 80°C with the relevant biochemical parameters determined accordingly. The tissue samples were frozen in liquid nitrogen or fixed in 4% buffered formalin for histopathological evaluation.

### 2.5 Serum biochemistry analysis

Determination of levels of aspartate aminotransferase (AST) and alanine aminotransferase (ALT) in serum samples and index for liver injury was performed using a clinical chemistry analyser system (VetTest 8008; IDEXX Laboratories). The level of lactate dehydrogenase (LDH‐3) and glutamate dehydrogenase (GLDH) in the respective serum samples were determined using a commercial kit (Nanjing Jiancheng Bioengineering Institute) in accordance with the manufacturer's instructions. In addition, the antioxidant defence markers including superoxide dismutase (SOD), glutathione S‐transferase (GST) and total antioxidant capacity (TAC) were evaluated based on previously reported literature methodology.^20^

### 2.6 Immunohistochemistry

Tissue samples were fixed by 10% formaldehyde, paraffin‐embedded and cut into 4‐μt serial sections. The tissue sections were heated at a temperature of 60°C in an oven for 1 hour, dewaxed using xylene, dehydrated by graded alcohol and incubated in 3% H_2_O_2_ (Sigma‐Aldrich Chemical Company) at 37°C for 30 minutes. After phosphate buffer saline (PBS) washing, the sections were immersed in citrate acid (0.01 mol/L) at 95°C and boiled for 20 minutes. After cooling to room temperature, the sections were rinsed with PBS, blocked with normal serum working solution at 37°C for 10 minutes and subsequently incubated with primary rabbit antimouse FOXO4 (ab63254, 1:200; Abcam Inc) at 37°C for 2 hours, followed by the addition of secondary antibody horseradish peroxidase (HRP)‐labelled goat anti‐rabbit immunoglobulin G (IgG) (ab6721, 1:1000; Abcam) at 37°C for 30 minutes. Sections were counterstained by haematoxylin (Shanghai Fusheng Industrial Co., Ltd.) for 4 minutes at room temperature, mounted using 10% glycerol/PBS and observed under a microscope. The results were evaluated by two independents observers in a double‐blinded fashion.

### 2.7 Haematoxylin‐eosin (HE) staining

Mouse liver tissues were paraffin‐embedded, sectioned and fixed with 10% neutral formaldehyde solution for more than 24 hours. After dewaxed twice using xylene (10 minutes each time), the sections were rehydrated by alcohol (100%, 90% and 70%) and stained by haematoxylin for 7 minutes. After washing under running water for 10 minutes, the sections were stained using eosin for 1 minute, rehydrated using gradient alcohol (100%, 95%, 75% and 50%) and cleared with xylene. The sections were air‐dried, mounted using balsam and observed under an optical microscope.

### 2.8 Terminal deoxynucleotidyl transferase‐mediated dUTP‐biotin nick end labelling (TUNEL) staining of liver tissues

The mouse liver tissues were fixed by 4% formaldehyde, paraffin‐embedded and cut into 5‐mm‐thick sections. The sections were dewaxed, diluted by 1% proteinase K (50 μL) and incubated at 37°C for 30 minutes, followed by the addition of 0.3% H_2_O_2_ at 37°C in order to eliminate endogenous peroxidase activity. The tissue sections were incubated with TUNEL reaction solution at 37°C for 1 hour in void of light. Next, the tissue sections were further incubated with 50 μL converter‐peroxidase at 37°C for 30 minutes in a humid box, developed using 2% diaminobenzidine, and incubated at room temperature for 15 minutes. The sections were subsequently observed under a microscope, and after the nuclei were observed to be brownish‐yellow, the sections were counterstained with haematoxylin, dehydrated, transparentized, mounted and then observed under an optical microscope (40×) with 10 fields of view randomly selected. Brownish‐yellow nuclei were considered as positive‐apoptotic cells. The cells with blue nuclei were deemed to be normal with the average value determined accordingly. The ratio of the number of yellow‐brown cells to that of blue cells was regarded as the rate of cell apoptosis.

### 2.9 TUNEL staining of cell apoptosis

The cells were dewaxed in xylene, rehydrated and treated with 20 μg/mL of proteinase K without DNase (recommended 20 mg/mL proteinase K, ST532/ST533; Beyotime Biotechnology Co., diluted 1000 folds with P0106 immunostaining washing solution or 10 mmol/L Tris‐HCl [pH 7.4‐7.8], namely 20 μg/mL proteinase K without DNase), at 20‐37°C for 15‐30 minutes. After three PBS or Hank's balanced salt solution washes, an appropriate amount of TUNEL detection solution was added to the cells and incubated at 37°C for 60 minutes under conditions void of light. Finally, the cells were blocked with fluorescence decay‐resistant medium and observed under a fluorescence microscope.

### 2.10 Primary hepatocyte isolation and treatment

Primary hepatocytes were isolated from the mouse liver tissues and cells with a viability greater than 90% were employed for further H/R experimentation.^21^ The medium was replaced with serum‐free Dulbecco's modified Eagle's medium, with the cells incubated under hypoxic conditions (1% O_2_, 5% CO_2_, and 94% N_2_) in a modular incubator (Biospherix), followed by reoxygenation (air/5% carbon dioxide) at the designated time‐points to simulate the hepatic I/R injury in vitro. The cells and the related medium were collected for further experiments.

### 2.11 Cell transfection

Primary hepatocytes exhibiting logarithmic growth were seeded into 6‐well plates at a density of 4 × 10^5^ cells/well. Confluent cells were transfected with plasmids of miR‐96 mimic, miR‐96 inhibitor, overexpression (oe)‐FOXO4 or their corresponding NCs, alone or in combination, as per the provided instructions of the Lipofectamine 2000 reagents (11668‐019; Invitrogen). The transfection sequences and plasmids were commercially obtained from GenePharma.

### 2.12 RNA isolation and quantification

Total RNA was extracted using TRIzol reagents (15596026; Invitrogen), which was reverse transcribed into complementary DNA in accordance with the manufacturer's instructions of the reverse transcription kit (RR047A; Takara). The samples were loaded using the SYBR Premix EX Taq kit (RR420A; Takara). Reverse transcription quantitative polymerase chain reaction (RT‐qPCR) was conducted using an ABI 7500 instrument (Applied Biosystems), which was conducted in triplicate. The primers for miR‐96, FOXO4 and glyceraldehyde‐3‐phosphate dehydrogenase (GAPDH) were synthesized by Sangon Biotech (Table 1). U6 was regarded as the internal control for miR‐96, whereas GAPDH was regarded as the internal control of the genes. The $2^{‐\Delta \Delta C_{t}}$ method was applied for RNA relative quantitation.

**Table 1:** Primer sequences for reverse transcription quantitative polymerase chain reaction

Target	Primer sequences (5′‐3′)
has‐miR‐96	F: GCCCGCTTTGGCACTAGCACATT
	R: GTGCAGGGTCCGAGGT
has‐FOXO4	F: CCGGCAAAAGCTCTTGGTG
	R: GGTCCACATATCGGCTTCTTCA
mmu‐miR‐96	F: CGGTTTGGCACTAGCACATT
	R: CAGTGCGTGTCGTGGAGT
mmu‐FOXO4	F: TGGGCTCAATCTCGCATCTC
	F: AAAGAGGGAGGCACCATAGC
mmu‐Bcl‐2	F: GTCCACGAACCCGTAAGGT
	F: CATCTTTTCCCGATAGGTCCA
mmu‐Bax	F: TGCTGCCTTTTCTGTTCCTT
	F: AAGGTGCTGGGTAGGGAAGT
mmu‐Fas	F: CTCCGAGTTTAAAGCTGAGG
	R: TGTACTCCTTCCCTTCTGTGC
U6	F: CGGCGGTCGTGAAGCGTTCCAT
	R: CCAGTGCAGGGTCCGAGGTAT
GAPDH	F: CACCCACTCCTCCACCTTTG
	R: CCACCACCCTGTTGCTGTAG‐3

Abbreviations: Bax, Bcl‐2‐associated X protein; Bcl‐2, B‐cell lymphoma 2; F, forward; FOXO4, forkhead box O4; GAPDH, glyceraldehyde‐3‐phosphate dehydrogenase; miR‐96, microRNA‐96; R, reverse.

### 2.13 Western blot analysis

Total protein was extracted from the tissues or cells via radio‐immunoprecipitation assay lysis buffer (R0010; Beijing Solarbio Science & Technology Co., Ltd.). The protein concentration of each sample was then measured using a bicinchoninic acid kit (20201ES76; Yeasen Biotechnological Co., Ltd). The protein was separated with sodium dodecyl sulphate‐polyacrylamide gel electrophoresis and subsequently transferred onto a polyvinylidene fluoride membrane, which was then blocked with 5% bovine serum albumin at room temperature for 1 hour. The membrane was incubated with diluted primary antibodies (Abcam Inc): rabbit polyclonal antibody to FOXO4 (ab63254, 1:1000), rabbit polyclonal antibody to FOXO4 (ab205921, 1:500) for different purpose, rabbit polyclonal antibody to B‐cell lymphoma 2 (Bcl‐2) (ab32124, 1:1000), rabbit anti‐human phosphorylated Bcl‐2‐associated X protein (Bax) (ab32503, 1:1000), rabbit polyclonal antibody to cleaved caspase‐3 (C‐casp3) (ab2302, 1:500) and rabbit polyclonal antibody to caspase‐3 (ab4051, 1:500) at 4°C overnight. HRP labelled goat anti‐rabbit IgG (ab205718, 1:10 000) was then added to the membrane under room temperature conditions for 1 hour. The image was developed using VILBER FUSION FX5 (Vilbert‐Lourmat). Quantitative analyses on the proteins were performed using ImageJ 1.48u software (National Institutes of Health), which was expressed as the ratio of the grey value of the target band to the internal reference band.

### 2.14 3‐(4,5‐dimethyl‐2‐thiazolyl)‐2,5‐diphenyl‐2H‐tetrazolium bromide (MTT) assay

Hepatocytes were incubated in the medium containing 10% foetal bovine serum to prepare cell suspension. The cells were then seeded into a 96‐well plate with 200 μL/well and incubated for 3‐5 days. Each well was then cultured with 20 μL MTT solution (5 mg/mL) for an additional 4 hours. The supernatant was removed after the suspended cells had been centrifuged. A total of 150 μL dimethyl sulfoxide was added to each well and oscillated for 10 minutes to melt the crystal well. Absorbance of each well was performed at a wavelength of 490 nm and measured using a standard microplate reader.

### 2.15 Immunofluorescence staining

Cell slides were fixed using formaldehyde for 15 minutes and washed three times with PBS with 3 minutes each time. The slides were subsequently penetrated with 0.5% Triton X‐100 (Sangon Biotech) at room temperature for 20 minutes, blocked with normal goat serum (Beijing Solarbio Science & Technology Co., Ltd.) at room temperature for 30 minutes, and respectively incubated with diluted primary antibody to rabbit antimouse proliferating cell nuclear antigen (PCNA) (1:100; ab18197; Abcam Inc) in a humid box overnight at 4°C. The diluted secondary antibody Alexa Fluor 647‐labelled donkey anti‐rabbit IgG (1:400; ab150075; Abcam Inc) was then added to the slides, which was incubated in a humid box at 37°C for 1 hour. The cells were then incubated with 4',6‐diamidino‐2‐phenylindole (BIODEE) under conditions void of light for 5 minutes for nuclei staining purposes. The cell slides were finally mounted using an anti‐fade mounting medium and analysed under a fluorescence microscope (Olympus).

### 2.16 Dual‐luciferase reporter gene assay

Wild‐type and mutant type (MUT) reporter plasmids contained in FOXO4 3′untranslated region were designed by GenePharma as pMIRGLO‐FOXO4‐WT and pMIRGLO‐FOXO4‐MUT, which were cotransfected with mimic NC or miR‐96 mimic into HEK293T cells over a period of 48 hours. After the cells had been collected, the luminescent signal representing the reporter gene activation was detected following the instructions of dual‐luciferase detection kit (D0010; Beijing Solarbio Science & Technology Co., Ltd.). The luminescent intensity was determined using the GLomax20/20 Luminometer (E5311; Shaanxi Zhongmei Biotechnological Co., Ltd).

### 2.17 Statistical analysis

All data analysed by SPSS 21.0 software (IBM Corp.) were expressed as mean ± SD. Data obeying normal distribution and homogeneity of variance between two groups were compared via an unpaired *t* test, whereas data between multiple groups were assessed by one‐way analysis of variance (ANOVA), followed by the application of a Tukey's test with corrections for multiple comparisons. Data at different time‐points were analysed using Bonferroni‐corrected repeated measures ANOVA. A value of *P < *.05 was considered to be indicative of statistical significance.

## 3 RESULTS

### 3.1 FOXO4 is highly expressed in hepatic I/R injury

**Figure 1:**
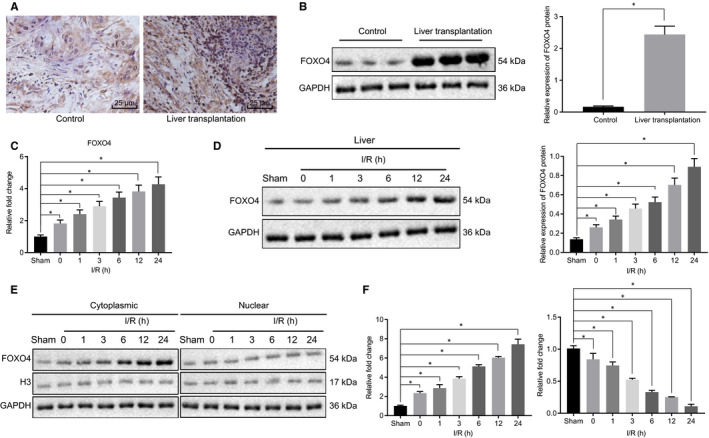
Increased expression of FOXO4 in hepatic I/R injury. A, The positive expression of FOXO4 protein in liver tissue samples of participants with liver transplantation and healthy donors detected by immunohistochemical staining. B, The protein expression of FOXO4 in liver tissue samples of participants with liver transplantation and healthy donors detected by Western blot analysis. *P < .05 compared with the liver tissue samples of healthy donors. C, The mRNA expression of FOXO4 determined by RT‐qPCR in liver tissues of mice treated with sham operation or I/R at different time‐points. *P < .05 compared with sham‐operated mice. D, The protein expression of FOXO4 determined by Western blot analysis in liver tissues of mice treated with sham operation or I/R at different time‐points. E, The expression of FOXO4 determined by Western blot analysis in the nucleus and cytoplasm of hepatocytes treated with H/R. F, The expression of Bax and Bcl‐2 determined by RT‐qPCR in liver tissues of mice treated with sham operation or I/R at different time‐points. *P < .05 compared with sham‐operated mice. The measurement data were expressed as mean ± SD. Data between two groups were analysed by unpaired *t* test. Data among multiple groups were analysed using one‐way ANOVA followed by Tukey's test. n = 12

In an attempt to elucidate the role of FOXO4 in hepatic I/R injury, the expression of FOXO4 in the clinical tissue samples of patients with liver transplantation was initially determined via immune‐histochemical staining and Western blot analysis. The results (Figure 1A,B) obtained demonstrated that in comparison to the control samples, the expression of FOXO4 was markedly up‐regulated in the clinical tissue samples of the liver transplantation patients. Meanwhile, the mRNA and protein expression of FOXO4 was found to be enhanced in the liver tissues of I/R mice in a time‐dependent manner when compared with that of the liver tissues of the sham‐operated mice (Figure 1C,D). The primary hepatocytes were isolated from human liver tissues and subsequently subjected to H/R. The Western blot analysis results revealed an elevated FOXO4 protein level in the nucleus and cytoplasm of the hepatocytes subjected to H/R (Figure 1E). The RT‐qPCR results depicted that the expression of FOXO4 downstream target genes had indeed been affected (Figure 1F). The aforementioned findings suggested that FOXO4 was expressed at high levels in hepatic I/R injury.

### 3.2 Sevo promotes miR‐96 expression and reduces FOXO4 expression and hepatocyte apoptosis providing an alleviatory effect in hepatic I/R injury in vivo

**Figure 2:**
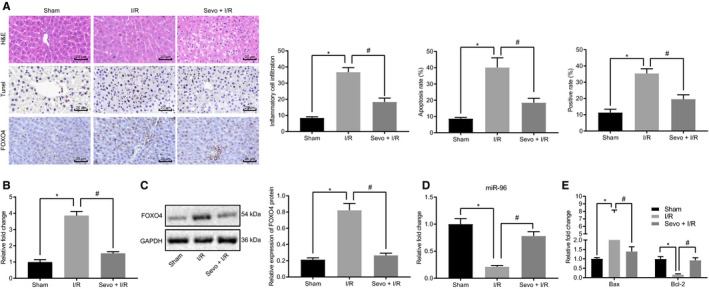
Sevo up‐regulates miR‐96 expression while inhibiting FOXO4 expression, and reducing hepatocyte apoptosis and liver injuries in vivo. A, The liver injury degree, hepatocyte apoptosis and FOXO4‐positive expression detected using HE staining, TUNEL staining and immunohistochemistry in liver tissues of I/R mice treated with Sevo. B, mRNA expression of FOXO4 determined by RT‐qPCR in liver tissues of I/R mice treated with Sevo. C, The protein expression of FOXO4 determined using Western blot analysis in liver tissues of I/R mice treated with Sevo. D, miR‐96 expression evaluated using RT‐qPCR in liver tissues of I/R mice treated with Sevo. E, The expression of Bax and Bcl‐2 determined by RT‐qPCR in liver tissues of I/R mice treated with Sevo. *P < .05 compared with sham‐operated mice. ^#^ *P* < .05 compared with the I/R mice. The measurement data were expressed as mean ± SD. Data between two groups were analysed by unpaired *t* test. Data among multiple groups were analysed using one‐way ANOVA followed by Tukey's test for n = 12

Next, to further evaluate the effect associated with Sevo and its associated regulatory mechanism on hepatic I/R injury, the mice were either anaesthetized using 2% Sevo or received sham operation or subjected to I/R. The results of HE staining showed that the liver tissues in sham‐operated mice were aligned orderly with normal form. However, in the mouse I/R model, severe injuries were detected in the liver tissues, accompanied by disorganized hepatocytes and denatured hepatocyte saccules when compared with the sham‐operated mice, whereas the injection of Sevo reduced the liver tissue injury following I/R (Figure 2A top).

The TUNEL staining results revealed that a greater number of apoptotic hepatocytes were present in the liver tissues of the I/R mice than in that of the liver tissues of sham‐operated mice. The I/R mice treated with Sevo exhibited a reduction in terms of the number of apoptotic hepatocytes in comparison to I/R mice (Figure 2A middle).

The immunohistochemistry results displayed an increase in the number of FOXO4‐positive cells in mice subjected to I/R, which decreased following Sevo treatment (Figure 2A below).

The relative expression of FOXO4 mRNA and protein was evaluated using Western blot analysis and RT‐qPCR (Figure 2B,C) which revealed that the liver tissues of the I/R mice were up‐regulated compared to sham‐operated mice, whereas Sevo treatment led to a downward trend. Besides, miR‐96 expression was down‐regulated in liver tissues of I/R mice in contrast to sham‐operated mice. However, miR‐96 expression was elevated in the liver tissues of I/R mice treated with Sevo compared with that of I/R mice (Figure 2D). FOXO4 downstream genes Bax and Bcl‐2 were determined by RT‐qPCR, the results of which revealed that there was an increase in the expression of Bax and a reduction in the expression of Bcl‐2 in the liver tissues of I/R mice than sham‐operated mice, whereas Sevo treatment brought about opposite effects (Figure 2E). The aforementioned results suggested that Sevo may enhance miR‐96 expression and reduce expression of FOXO4 and its downstream genes as well as attenuating hepatocyte apoptosis, and liver injury degree induced by I/R.

### 3.3 miR‐96 targets FOXO4 and negatively regulates its expression in vitro

**Figure 3:**
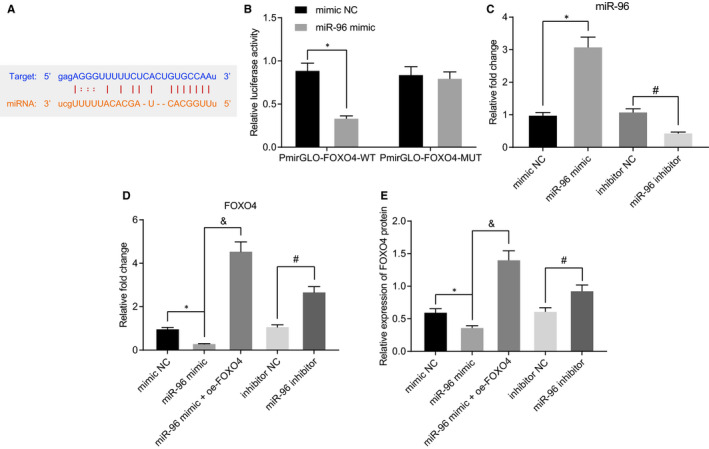
FOXO4 is a target gene of miR‐96. A, The predicted binding sites between miR‐96 and FOXO4 by bioinformatics analysis (http://starbase.sysu.edu.cn/). B, The binding of miR‐96 to FOXO4 verified using dual‐luciferase reporter gene assay in HEK293T cells transfected with miR‐96 mimic. *P < .05 compared with HEK293T cells transfected with mimic NC. C, The expression of miR‐96 in hepatocytes transfected with miR‐96 mimic or inhibitor detected by RT‐qPCR. D, The mRNA expression of FOXO4 assessed using RT‐qPCR in hepatocytes transfected with miR‐96 mimic, miR‐96 inhibitor or miR‐96 mimic + oe‐FOXO4. E, The protein expression of FOXO4 examined using Western blot analysis in hepatocytes transfected with miR‐96 mimic, miR‐96 inhibitor or miR‐96 mimic + oe‐FOXO4. *P < .05 compared with hepatocytes transfected with mimic NC. ^#^ *P* < .05 compared with hepatocytes transfected with inhibitor NC. &*P < *.05 compared with hepatocytes transfected with miR‐96 mimic. The measurement data were expressed as mean ± SD. Data between two groups were analysed by unpaired *t* test. Data among multiple groups were analysed using one‐way ANOVA followed by Tukey's test. The cellular experiment was repeated three times

The binding sites between miR‐96 and FOXO4 were predicted *via* bioinformatics analysis (Figure 3A), which was verified using dual‐luciferase reporter gene assay. As depicted in Figure 3B, the luciferase activity of pMIRGLO‐FOXO4‐WT was inhibited whereas that of pMIRGLO‐FOXO4‐MUT exhibited no significant changes in HEK293T cells transfected with miR‐96 mimic. Furthermore, the RT‐qPCR results illustrated that compared to transfection with mimic NC, transfection with miR‐96 mimic triggered a reduction in the expression of FOXO4 in the primary hepatocytes, whereas the expression of FOXO4 was enhanced following cotransfection with miR‐96 mimic and oe‐FOXO4. FOXO4 expression was also increased in the hepatocytes transfected with miR‐96 inhibitor relative to transfection with inhibitor NC. Western blot analysis revealed similar findings (Figure 3C‐E). The regulation of FOXO4 by miR‐96 showed the same trend in control mice (Figure S1). The aforementioned results provided evidence suggesting that miR‐96 targeted FOXO4.

### 3.4 Knockdown of FOXO4 relives hepatic I/R injury in vivo

**Figure 4:**
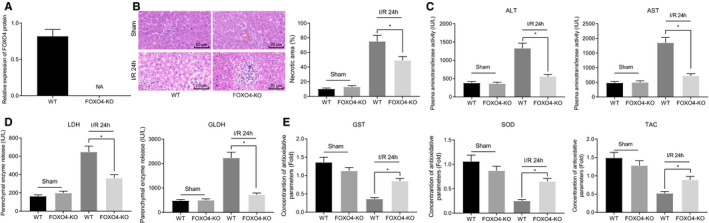
Knockdown of FOXO4 exerts protective function in hepatic I/R injury in vivo. A, The protein expression of FOXO4 examined using Western blot analysis in liver tissues of control and FOXO4‐KO mice. *P < .05 compared with the control mice subjected to sham operation. B, Liver tissue injury observed using HE staining. C, The serum levels of ALT and AST in control and FOXO4‐KO mice subjected to I/R for 24 h. D, The serum levels of LDH and GLDH in control and FOXO4‐KO mice subjected to I/R for 24 h. E, The serum levels of GST, SOD and TAC in control and FOXO4‐KO mice subjected to I/R for 24 h. *P < .05 compared with the control mice subjected to sham operation or I/R for 24 h. The measurement data were expressed as mean ± SD. Data between two groups were analysed by unpaired *t* test. Data among multiple groups were analysed using one‐way ANOVA followed by Tukey's test. n = 12

A marked increase in the expression of FOXO4 was identified during hepatic I/R injury after which we suggested that FOXO4 is a crucial component in the setting of hepatic I/R injury. Hence, we constructed FOXO4‐KO mouse models to further elucidate its role. The results of Western blot analysis exhibited that FOXO4 expression was successfully knocked down in the liver tissues of the FOXO4‐KO mice when compared to the control mice (Figure 4A). The injury of liver tissues was evaluated using HE staining, which (Figure 4B) revealed a large area of necrosis in liver tissues of FOXO4‐KO mice subjected to I/R for 24 hours compared with control mice subjected to I/R for 24 hours. Correspondingly, the serum levels of ALT, AST, LDH and GLDH were all diminished, whereas those of SOD, GST and TAC were elevated in FOXO4‐KO mice subjected to I/R for 24 hours compared with control mice subjected to I/R for 24 hours (Figure 4C‐E). Altogether, the above results suggested that deficient FOXO4 could protect against hepatic I/R injury in vivo.

### 3.5 Knockdown of FOXO4 inhibits apoptosis but promotes proliferation of hepatocytes in vitro

**Figure 5:**
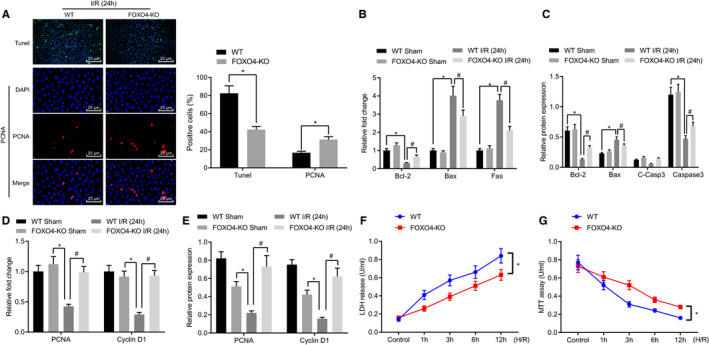
Absence of FOXO4 inhibits cell apoptosis but promotes cell proliferation in vitro. Primary hepatocytes were isolated from the liver tissues of mice in each group and treated with H/R to simulate the hepatic I/R injury model in vitro. A, TUNEL and immunofluorescence staining analyses of H/R‐treated hepatocytes from FOXO4‐KO mice. *P < .05 compared with hepatocytes isolated from control mice. B, The mRNA expression of cell apoptosis‐related genes (Bax, Fas, Bcl‐2 and C‐Casp3) detected using RT‐qPCR in H/R‐treated hepatocytes from FOXO4‐KO mice. *P < .05 compared with the hepatocytes isolated from control mice. ^#^ *P* < .05 compared with the hepatocytes isolated from control mice subjected to I/R for 24 h. C, The protein expression of cell apoptosis‐related genes (Bax, Fas, Bcl‐2 and C‐Casp3) detected using Western blot analysis in H/R‐treated hepatocytes from FOXO4‐KO mice. *P < .05 compared with the hepatocytes isolated from control mice. ^#^ *P* < .05 compared with the hepatocytes isolated from control mice subjected to I/R for 24 h. D, The mRNA expression of cell proliferation‐related genes (PCNA and Cyclin D1) measured using RT‐qPCR in H/R‐treated hepatocytes from FOXO4‐KO mice. *P < .05 compared with the hepatocytes isolated from control mice. ^#^ *P* < .05 compared with the hepatocytes isolated from control mice subjected to I/R for 24 h. E, The protein expression of cell proliferation‐related genes (PCNA and Cyclin D1) assessed using Western blot analysis in H/R‐treated hepatocytes from FOXO4‐KO mice. *P < .05 compared with the hepatocytes isolated from control mice. ^#^ *P* < .05 compared with the hepatocytes isolated from control mice subjected to I/R for 24 h. F, Measurement of LDH levels in H/R‐treated hepatocytes from FOXO4‐KO mice. *P < .05 compared with the control mice. G, Cell viability measured by MTT assay in H/R‐treated hepatocytes from FOXO4‐KO mice. *P < .05 compared with the hepatocytes isolated from control mice. The measurement data were expressed as mean ± SD. Data between two groups were analysed by unpaired *t* test. Data among multiple groups were analysed using one‐way ANOVA followed by Tukey's test. The cellular experiment was repeated three times

Next, in order to further observe the effect of FOXO4 on hepatocyte apoptosis and proliferation, primary hepatocytes were isolated from the liver tissues of mice in each group and treated with H/R to simulate the hepatic I/R injury model in vitro. Analyses on the isolated primary hepatocytes using immunofluorescence and TUNEL staining demonstrated that relative to the hepatocytes from the control mice subjected to I/R for 24 hours, the hepatocytes from FOXO4‐KO mice subjected to I/R for 24 hours exhibited a lesser degree cell apoptosis (TUNEL staining: positive) and more cell proliferation (PCNA: positive) (Figure 5A), which was accompanied by an elevated expression of anti‐apoptotic gene Bcl‐2 and decreased mRNA expression of apoptotic gene Bax and Fas (Figure 5B). When compared with the hepatocytes of the control mice subjected to I/R for 24 hours, the protein expression of pro‐apoptosis factors Bax and C‐Casp3 was reduced in hepatocytes from FOXO4‐KO mice subjected to I/R for 24 hours (Figure 5C). Furthermore, the expression of the hepatocyte proliferation‐related genes (PCNA and cyclin D1) was enhanced in hepatocytes isolated from FOXO4‐KO mice subjected to I/R for 24 hours (Figure 5D,E). In addition, a lower level of LDH detected in the hepatocytes from FOXO4‐KO mice subjected to I/R for 24 hours than that in hepatocytes from the control mice subjected to I/R for 24 hours (Figure 5F). The MTT assay results indicated an enhancement in the relation to the viability of hepatocytes from the FOXO4‐KO mice subjected to I/R for 24 hours (Figure 5G). The aforementioned findings suggested that loss of FOXO4 could promote cell proliferation while reducing cell apoptosis during hepatic I/R in vitro.

### 3.6 Sevo‐mediated miR‐96 promotion abates hepatic I/R injury in vivo

**Figure 6:**
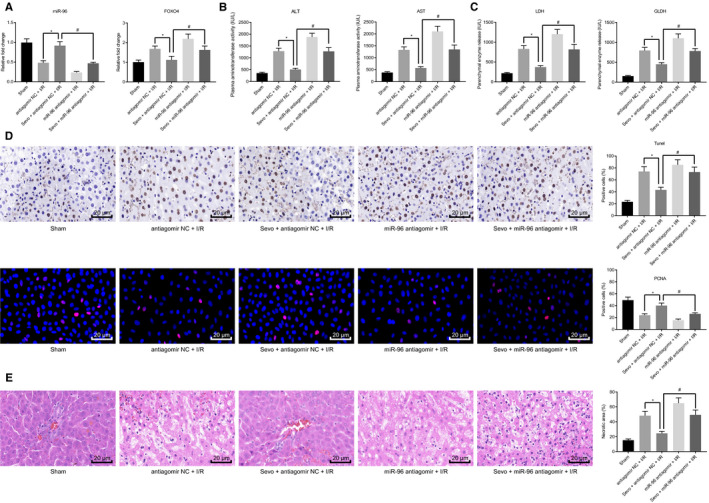
Sevo increases miR‐96 expression and protects the liver against hepatic I/R injury in vivo. A, The expression of FOXO4 and miR‐96 detected using RT‐qPCR in liver tissues of I/R mice treated with Sevo or in combination with miR‐96 antagomir. B, The serum levels of ALT and AST determined using biochemical analysis in I/R mice treated with Sevo or in combination with miR‐96 antagomir. C, The serum levels of LDH and GLDH determined using biochemical analysis in I/R mice treated with Sevo or in combination with miR‐96 antagomir. D, TUNEL and immunofluorescence staining of liver tissues of I/R mice treated with Sevo or in combination with miR‐96 antagomir. E, HE staining of liver tissue necrosis in I/R mice treated with Sevo or in combination with miR‐96 antagomir. *P < .05 compared with I/R mice treated with antagomir NC. ^#^ *P* < .05 compared with I/R mice treated with Sevo + antagomir NC. The measurement data were expressed as mean ± SD. Data among multiple groups were analysed using one‐way ANOVA followed by Tukey's test. n = 12

Reverse transcription quantitative polymerase chain reaction was performed to determine the expression of miR‐96 and FOXO4 in liver tissues of the mice following various treatments. The results displayed that compared with the I/R mice treated with antagomir NC, the expression of miR‐96 was increased, whereas FOXO4 expression was decreased in liver tissues of I/R mice after treatment with Sevo + antagomir NC. However, a contrasting trend was identified following treatment with Sevo + miR‐96 antagomir (Figure 6A). The biochemical analysis results demonstrated that in contrast to the I/R mice treated with antagomir NC, serum levels of ALT, AST, LDH and GLDH were decreased in the I/R mice treated with Sevo + antagomir NC, but Sevo + miR‐96 antagomir treatment brought about increased serum levels (Figure 6B,C). As illustrated in Figure 6D, lower hepatocyte apoptosis rate was identified in the liver tissues of I/R mice treated with Sevo + antagomir NC in contrast to that of I/R mice treated with antagomir NC, which was increased following treatment with Sevo + miR‐96 antagomir. Moreover, HE staining analysis suggested that liver necrosis was improved following Sevo treatment, with a large area of necrosis in the liver tissues detected following further treatment with miR‐96 antagomir (Figure 6E). The aforementioned results suggested that inhibition of miR‐96 could abolish the protective effect of Sevo against hepatic I/R injury in mice.

### 3.7 miR‐96 mitigates hepatic I/R injury by suppressing FOXO4 in vivo

**Figure 7:**
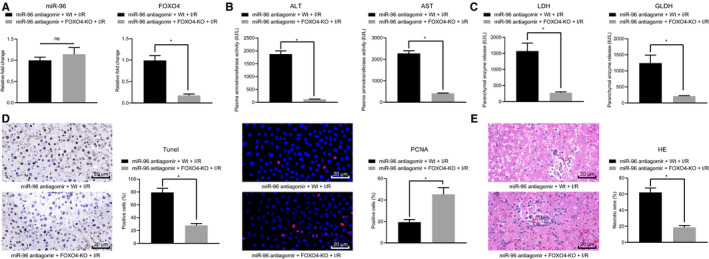
miR‐96 prevents hepatic I/R injury *via* FOXO4 suppression in vivo. A, The expression of FOXO4 and miR‐96 detected using RT‐qPCR in liver tissues of FOXO4‐KO mice treated with miR‐96 antagomir. B, The serum levels of ALT and AST determined using biochemical analysis in FOXO4‐KO mice treated with miR‐96 antagomir. C, The serum levels of LDH and GLDH determined using biochemical analysis in FOXO4‐KO mice treated with miR‐96 antagomir. D, TUNEL and PCNA immunofluorescence staining of liver tissues of FOXO4‐KO mice treated with miR‐96 antagomir. E, HE staining of liver tissue necrosis in FOXO4‐KO mice treated with miR‐96 antagomir. *P < .05 compared with control mice treated with miR‐96 antagomir. The measurement data were expressed as mean ± SD. Data among multiple groups were analysed using one‐way ANOVA followed by Tukey's test. n = 12

Finally, we set out to ascertain whether the protective effect of Sevo against hepatic I/R injury correlates to the miR‐96 target gene FOXO4. Sevo was employed to anaesthetize the FOXO4‐KO mice for hepatic I/R injury model establishment purposes, after which the mice were injected with miR‐96 antagomir *via* tail vein. The RT‐qPCR results revealed no notable changes in regard to the expression of miR‐96, whereas the expression of FOXO4 was reduced in liver tissues of control and FOXO4‐KO mice upon miR‐96 antagomir treatment (Figure 7A). Besides, biochemical analysis revealed that the serum levels of ALT, AST, LDH and GLDH were markedly higher in the FOXO4‐KO mice treated with miR‐96 antagomir in contrast to the control mice treated with miR‐96 antagomir (Figure 7B,C). As shown in Figure 7D, immunofluorescence and TUNEL assays showed lower hepatocyte apoptosis rate and higher PCNA expression in the liver tissues of FOXO4‐KO mice treated with miR‐96 antagomir than control mice treated with miR‐96 antagomir. Furthermore, HE staining analysis suggested that liver necrosis was alleviated in FOXO4‐KO mice treated with miR‐96 antagomir (Figure 7E). Altogether, the results obtained indicated that miR‐96 exerted protective effects against hepatic I/R injury in mice by down‐regulating FOXO4.

## 4 DISCUSSION

Hepatic I/R injury is often characterized by hepatocyte death as well as severe inflammation.^22^ Hepatic I/R has been well documented to have a notable negative impact on the outcome of liver surgery, highlighting the need to further investigate ischaemic preconditioning on hepatic I/R.^23^ Sevo is a frequently employed agent in clinical practice as an inhaled anaesthesia agent that has been reported to exert a protective role in hepatic I/R injury.^24^ Nevertheless, the underlying molecular mechanism by which this occurs is still under investigation. The present study set out to elucidate the role of Sevo from a molecular mechanism point of view in hepatic I/R injury. The results obtained indicated that Sevo could potentially arrest hepatocyte apoptosis and alleviate hepatic I/R injury by promoting miR‐96 expression and suppressing FOXO4 expression.

Initially, our results demonstrated that FOXO4 was expressed at comparatively higher levels in the liver tissues collected from patients that underwent liver transplantation, I/R mice and hepatocytes subjected to H/R. FOXO proteins represent a class of transcriptional factors that interact with DNA, whereby together with FOXO4 as well as three other FOXO proteins have been implicated in regulation of cell apoptosis and the cell cycle.^25^ Moreover, previous studies have emphasized FOXO4 as a contributory factor in a wide array of diverse liver diseases including that of alcohol‐induced liver disease, whereby it plays a role in hepatic inflammation.^26^ In line with our findings, FOXO4 found to be aberrantly activated in both hepatocellular carcinoma tissues and cell lines, functions as an oncogene in this cancer and promotes cell proliferation while attenuating apoptosis.^27^

We subsequently identified that the knockdown of FOXO4 could inhibit hepatocyte apoptosis but promote hepatocyte proliferation in hepatic I/R injury to prevent hepatic I/R injury in vivo. Consistent with the findings of the current study, previous reports have suggested that depletion of FOXO4 expression could protect pro‐angiogenic cells against apoptosis induced by oxidative stress by means of reducing the expression of C‐casp3 in ischaemic limbs.^28^ Notably, the knockdown of FOXO4 has been demonstrated to attenuate doxorubicin‐induced apoptosis in normal liver cells.^29^ Furthermore, proliferative and anti‐apoptotic characteristics are often strongly correlated with alleviation of hepatic I/R injury.^30^ Moreover, the hepatic antioxidant defence markers (SOD, GST and TAC) as well as serum level indexes such as ALT, AST, LDH and GLDH have also been highlighted. Ischaemic preconditioning is able to influence the level of antioxidant defence enzymes in hepatic I/R rats.^31^ Coincidentally, the application of Sevo has been shown to decrease liver serum markers such as ALT, AST, LDH and GLDH, whereas the expression of antioxidant defence markers was elevated all of which was consistent with the observations made in the current study.^7^

The results of the present study further illustrated that Sevo promoted the expression of miR‐96 while contributing to a decrease in the expression of FOXO4 leading to a reduction in the degree of injury induced by hepatic I/R. Sevo has been highlighted to reduce hepatic I/R injury in rats by combining the janus kinase 2/signal transducer and activator of transcription‐3‐pathway.^32^ The protective role of Sevo in hepatic I/R injury has been supported in existing literature, which has suggested that Sevo works in tandem with hepatic stellate cells.^33^ In line with the findings of the current study, a previous report concluded that Sevo exerts protective function in hepatic I/R injury,^34^ which could promote the expression of miR‐96,^35^ though the details of the relevant regulatory mechanism is yet to be fully elucidated. Moreover, Sevo has been suggested to markedly diminish hepatocyte apoptosis by suppressing the glucose regulatory protein 78.^36^

The bioinformatics analysis results (ENCORI website http://starbase.sysu.edu.cn/) suggested that miR‐96 targeted FOXO4, which was verified by the dual‐luciferase reporter gene assay. As evidenced by existing literature, miR‐29c and miR‐132 have been shown to target FOXO and down‐regulate its expression in alcoholic hepatitis.^37^ Overexpressed miR‐142‐5p negatively regulates FOXO expression to suppress cancer cell growth and alleviate hepatocellular carcinoma.^38^ However, to our knowledge limited studies have reported the targeting relation between miR‐96 and FOXO4 before.

## 5 CONCLUSION

**Figure 8:**
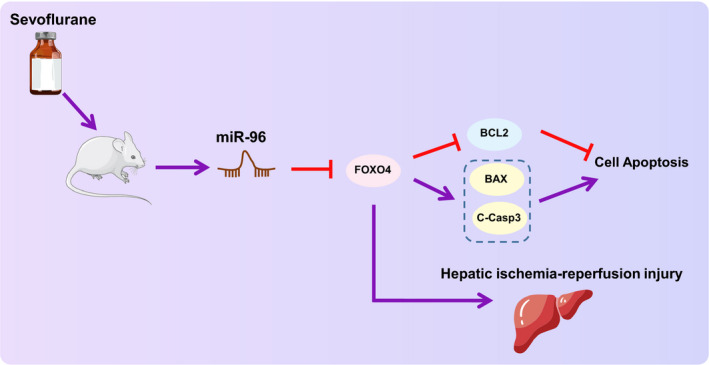
Molecular mechanism diagram depicting the role of Sevo in hepatic I/R injury. Sevo up‐regulates expression of miR‐96 and then suppresses the expression of miR‐96 target FOXO4, thereby reducing hepatocyte apoptosis and attenuating hepatic I/R injury

In conclusion, our study presents evidence demonstrating that Sevo increased miR‐96 expression causing the down‐regulation of the miR‐96 target FOXO4, ultimately accelerating hepatocyte proliferation and repressing hepatocyte apoptosis to attenuate hepatic I/R injury (Figure 8). The key findings of the current study provide a foundation for future understanding of the role of Sevo‐based molecular mechanism on regulating hepatocyte biological function as well as hepatic I/R injury.

## CONFLICT OF INTEREST

The authors declare that they have no competing interests.

## AUTHOR CONTRIBUTIONS

**Binghua He:** Data curation (equal); Investigation (equal); Supervision (equal); Writing‐original draft (equal). **Fan Yang:** Conceptualization (equal); Methodology (equal); Resources (equal). **Yingxia Ning:** Data curation (equal); Formal analysis (equal); Investigation (equal). **YaLan Li:** Conceptualization (equal); Methodology (equal); Resources (equal); Writing‐review & editing (equal).

## ETHICS APPROVAL

The current study was conducted with the approval of the Ethics Committee of the First Affiliated Hospital of Jinan University and performed in strict accordance with the *Declaration of Helsinki*. Signed informed consents were obtained from all participants or their family members prior to sample collection. All animal experiments were performed in strict accordance with the *Guide for the Care and Use of Laboratory Animal* published by the US National Institutes of Health and authorized by the experimental animal committee of the First Affiliated Hospital of Jinan University. Extensive efforts were made to ensure minimal suffering of the included animals.

## Supporting information

Fig S1

## Data Availability

Research data are not shared.
